# Association Between Airway Stenosis Degree and Respiratory Distress in Infants With a Vascular Ring

**DOI:** 10.7759/cureus.47022

**Published:** 2023-10-14

**Authors:** Koji Nakae, Kentaro Ueno, Yasuhiro Okamoto

**Affiliations:** 1 Pediatrics, Kagoshima University Hospital, Kagoshima, JPN

**Keywords:** heart diseases, dyspnea, airway stenosis, computed tomography, vascular ring

## Abstract

Background

Although the number of cases of prenatally diagnosed vascular rings is increasing, some cases may remain asymptomatic, and no indicator of the appearance of dyspnea has been established. Thus, we aimed to determine the relationship between the degree of airway compression by the vascular ring on contrast-enhanced computed tomography (CT) and respiratory distress.

Methods

This is a retrospective study of nine patients diagnosed with vascular rings at a single hospital from July 2010 to December 2019. Data regarding the patient's clinical characteristics, such as prenatal diagnosis, vascular ring type, complicated cardiac disease, and presence or absence of surgery, were recorded. Airway assessment on contrast-enhanced CT was measured in the axial cross-section. Statistical analysis was performed using Statistical Product and Service Solutions (SPSS) (version 25.0; IBM SPSS Statistics for Windows, Armonk, NY).

Results

Five of the eight patients had respiratory distress. Patients with respiratory distress were less likely to have been diagnosed prenatally (p = 0.04) and had smaller stenosis degree of anteroposterior diameter (p = 0.03).

Conclusion

Contrast-enhanced CT is useful in patients with vascular rings. Our study suggests that the stenosis degree of the anterior-posterior diameter of the airway is related to dyspnea.

## Introduction

The vascular ring is a rare malformation of thoracic-derived vascular and ligamentous structures, accounting for approximately 1% of congenital cardiovascular anomalies [1]. The condition results during the abnormal development of the aortic arch, and respiratory and gastrointestinal symptoms are caused when the bronchus trachealis or esophagus, or both, are surrounded by the aberrantly configured arch and/or associated vessels [2-4]. The most common form of vascular ring is a single aortic arch consisting of the right aortic arch, abnormal left subclavian artery, and left arterial ligament. A mirror-image single aortic arch (i.e., left aortic arch, anomalous right subclavian artery, and anomalous right arterial ligament) occurs much less frequently. The second most-common vascular ring is the double aortic arch [5]. Vascular ring classification was established by Edwards et al. [6] on the basis of their functioning double aortic arch system. With the increasingly widespread use of prenatal diagnosis [7], vascular ring is sometimes diagnosed prenatally with other cardiac malformation complications, but there are cases that remain asymptomatic. However, if not diagnosed prenatally, there are many differential diagnoses of dyspnea and dysphagia in children, and a definitive vascular ring diagnosis is often made after extensive investigation. Contrast-enhanced computed tomography (CT) is useful in the morphologic diagnosis of vascular rings, but the evaluation of respiratory distress is not well established, and no study has evaluated the degree of airway compression and respiratory impairment caused by vascular rings with contrast-enhanced CT [8]. Here, we aimed to clarify the association between the degree of airway compression at the vascular ring and respiratory distress on contrast-enhanced CT.

## Materials and methods

Ethical statements

The Kagoshima University Ethics Committee approved this study, and it was performed following the International Conference on Harmonization Good Clinical Practice Guidelines and the Declaration of Helsinki. All procedures involving human participants were in accordance with the ethical standards of the institutional and/or national research committee. The requirement for parental- and patient-informed consent was waived because of the retrospective nature of this study.

Study design and patients

We conducted a retrospective observational study of nine patients with a diagnosis of vascular ring at our hospital from July 2010 to December 2019 using their medical records. Eight of the patients enrolled in the study underwent contrast-enhanced CT, but one patient was asymptomatic and did not undergo contrast-enhanced CT due to a policy of careful follow-up and was excluded. Additionally, controls were established to evaluate the airway measurements in patients without vascular rings and respiratory distress. The controls comprised 10 patients with tetralogy of Fallot without airway compromise who underwent contrast-enhanced CT in infancy before cardiac surgery.

Demographic and clinical data

We examined the association between the frequency of respiratory distress and the degree of airway narrowing on contrast-enhanced CT in patients diagnosed with vascular rings. The definition of respiratory distress in this study was the presence of breathing effort or noisy breathing (wheezing and/or stridor), and the need for oxygen or positive pressure ventilation. Patient clinical characteristics such as age, gender, weight, prenatal diagnosis, vascular ring type, combined cardiac disease, surgical procedures, etc. were retrospectively examined. Vascular ring morphology was classified using the Edwards classification.

Evaluation of the airway in contrast-enhanced computed tomography

**Figure 1:**
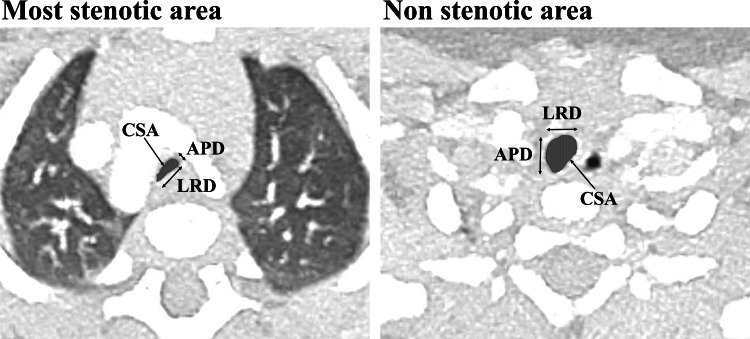
Airway measurement methods Lumen diameters (APD, LRD, CSA) were measured in axial cross-section in the pulmonary window setting of contrast-enhanced computed tomography. The tracheal lumen is indicated by vertical stripes. APD, anterior-posterior diameter; CSA, cross-sectional area; LRD, left-right diameter

Contrast CT was performed with patients sedated but still spontaneously breathing, with iopamidol as the contrast agent. Helical CT slice thickness was 1 mm without electrocardiographic synchronization. The axial cross-section was used in a pulmonary window setting to evaluate the airway, and the lumen diameter was measured according to the method presented by Griscom et al. [9] (Figure 1). Measurements were taken manually using a mouse on the electronic medical record. We measured the anterior-posterior diameter (APD), left-right diameter (LRD), and cross-sectional area (CSA) of the most stenotic and non-stenotic areas of the airway, respectively. As the most stenotic area is affected by the direction of compression by the vascular ring, APD is defined as the short axis diameter, and LRD is the long axis diameter in the most stenotic area (Figure 1). The non-stenotic area was defined as 10 mm headward from the most stenotic area. The stenosis degree in the anterior-posterior diameter (SD-APD) was defined as the narrowest APD divided by the non-stenotic APD, in the left-right diameter was defined as the narrowest LRD divided by the non-stenotic LRD, and in the cross-sectional area was defined as the narrowest CSA divided by the non-stenotic CSA. The most stenotic area of the controls was just above the tracheal bifurcation, as the same area is often stenotic in the vascular ring. The non-stenotic area of the control was the same as in the vascular ring cases.

Outcomes

The primary endpoint was to determine the relationship between the degree of airway compression and respiratory distress on contrast-enhanced CT in patients with the vascular ring. Secondary endpoints included prenatal diagnosis, complications of cardiac malformations, and genetic and chromosomal abnormalities in patients with vascular ring.

Statistical analysis

Continuous variables were expressed as median and interquartile range (IQR, 25th-75th percentile) and categorical variables as frequencies and percentages. Comparisons of measurements in the presence or absence of respiratory distress were made using the Mann-Whitney U test. Comparisons of each airway measurement between the three groups including control were made using the Kruskal-Wallis test and the post hoc test (nonparametric: Steel-Dwass test). Statistical significance was set at p < 0.05. All statistical analyses were performed using Statistical Product and Service Solutions (SPSS) (version 25.0; IBM SPSS Statistics for Windows, Armonk, NY).

## Results

Clinical characteristics of patients with a vascular ring

Four patients (50%) were diagnosed prenatally, and five (63%) had respiratory distress, as shown in Table 1. Three patients who underwent CT without respiratory distress were all prenatal diagnosis cases: two for the preoperative evaluation of congenital heart disease, and one was admission to nursery owing to some parents being high social risks because of undergoing postnatal diagnosis before discharge. In terms of vascular ring classification, IIIB1 (right aortic arch, left subclavian artery anomaly, left ductus arteriosus) was the most common (six patients; 75%). Kommerell diverticulum was identified in all cases except Case 1, which was a double aortic arch. Five patients (63%) had congenital heart disease, and all had a ventricular septal defect. Seven patients (88%) underwent surgery for vascular rings. Genetic abnormalities were observed in four patients (50%), all with 22q11.2 deletion syndrome. None of the patients had a congenital tracheal ring complication. The airway measurement data for the eight patients who underwent contrast-enhanced CT are shown in Table 1.

**Table 1:** Clinical characteristics of patients with vascular ring and airway measurements on computed tomography images APD, anterior-posterior diameter; BSA, body surface area; CHD, congenital heart disease; CSA, cross-sectional area; CT, computed tomography; LRD, left-right diameter

Case		1	2	3	4	5	6	7	8
Clinical characteristics									
Age at diagnosis (day)		154	0	0	0	39	1	0	0
Height (cm)		65	48	57	76	54	45	70	56
Body weight (kg)		7.3	2.7	4.9	10	3.1	1.9	7.4	4.8
Sex (M, Male; F, Female)		M	F	M	F	F	M	F	F
Prenatal diagnosis		No	Yes	Yes	Yes	No	No	Yes	No
Age at CT (day)		154	8	128	386	40	4	308	86
Respiratory distress		Yes	No	No	Yes	Yes	Yes	No	Yes
Vascular ring type		ⅠA_1_	ⅢB_1_	ⅢB_1_	ⅢB_1_	ⅢB_1_	ⅢB_1_	ⅢB_1_	ⅡB_2_
Complication of CHD		No	No	Yes	No	Yes	Yes	Yes	Yes
Age at surgery (month)		5	-	5	14	1	5	13	2
Genetic abnormality		No	No	Yes	No	Yes	Yes	Yes	No
Airway measurements									
Most stenotic area									
	APD (cm)	1.2	3.1	4.1	3.8	2.5	1.9	2.6	1.2
	LRD (cm)	4.3	7.1	8.7	14	6	7.4	7.1	5.7
	APD/LRD	0.3	0.4	0.5	0.3	0.4	0.3	0.4	0.2
	CSA (cm^2^)	4.1	17	28	42	12	11	14	5.4
	CSA/BSA (cm^2^/m^2^)	11	91	101	91	54	72	38	20
Non-stenotic area									
	APD (cm)	6.2	5.1	6.2	6.4	4.2	4.2	4.1	3.5
	LRD (cm)	6.9	5.4	7.8	9.4	4.9	3.6	7	3.5
	APD/LRD	0.9	0.9	0.8	0.7	0.9	1.2	0.6	1
	CSA (cm^2^)	34	22	38	47	16	12	23	9.6
	CSA/BSA (cm^2^/m^2^)	93	114	137	103	75	78	59	35
Stenosis degree									
	APD	0.19	0.61	0.66	0.59	0.59	0.45	0.63	0.34
	LRD	0.62	1.3	1.1	1.5	1.2	2.1	1	1.6
	CSA	0.12	0.8	0.74	0.88	0.73	0.93	0.64	0.56

Comparison between with and without respiratory distress among patients with a vascular ring

Five patients with and three without respiratory distress were included in the study (Table 2). Prenatal diagnosis was performed in all patients without respiratory distress, but only in one (20%) with respiratory distress (p = 0.04). Surgery was performed in all patients with and two (67%) without respiratory distress (p = 0.20). There was no clear difference between patients with and without respiratory distress in terms of congenital heart disease complications and genetic abnormalities. Regarding airway measurements on contrast-enhanced CT, patients with respiratory distress had a significantly smaller SD-APD than those without respiratory distress (0.5 (0.3-0.6) vs 0.6 (0.6-0.7); p = 0.03). There were no clear differences in LRD or CSA measurements and stenosis degree between patients with and without respiratory distress.

**Table 2:** Comparison of cases with and without respiratory distress Data are expressed as median values with interquartile range (25th–75th percentiles) or as a number (proportion, %). Bold font indicates significant p values. APD, anterior-posterior diameter; BSA, body surface area; CHD, congenital heart disease; CSA, cross-sectional area; CT, computed tomography; LRD, left-right diameter

	Respiratory distress (n=5)	Non-respiratory distress (n=3)	P-value
Clinical characteristics			
Age at diagnosis (day)	0 (0–1)	0 (0–0)	0.12
Height (cm)	56 (54–65)	57 (52–64)	0.88
Body weight (kg)	4.8 (3.1–7.3)	4.9 (3.8–6.1)	0.88
Sex (Male)	2 (40%)	1 (33%)	0.77
Prenatal diagnosis	1 (20%)	3 (100%)	0.04
Age at CT (day)	86 (40–154)	128(68–218)	0.88
Complication of CHD	3 (60%)	2 (67%)	0.86
Surgery	5 (100%)	2 (67%)	0.20
Age at surgery (month)	5 (2–5)	9 (7–11)	0.42
Genetic abnormality	2 (40%)	2 (67%)	0.50
Airway stenosis			
Most stenotic area			
APD (cm)	1.9 (1.2–2.5)	3.1 (2.9–3.6)	0.10
LRD (cm)	6.0 (5.7–7.4)	7.1 (7.1–7.9)	0.45
APD/LRD	0.3 (0.3–0.3)	3.1 (2.9–3.6)	0.05
CSA (cm^2^)	11 (5.4–12)	17 (16–23)	0.18
CSA/BSA (cm^2^/m^2^)	54 (20–72)	91 (65–96)	0.30
Non-stenotic area			
APD (cm)	4.2 (4.2–6.2)	5.1 (4.6–5.7)	1.0
LRD (cm)	4.9 (3.6–6.9)	7.0 (6.2–7.4)	0.30
APD/LRD	0.9 (0.9–1.0)	0.8 (0.7–0.9)	0.30
CSA (cm^2^)	16 (12–34)	22 (22–30)	0.46
CSA/BSA (cm^2^/m^2^)	78 (75–93)	114 (87–126)	0.30
Stenosis degree			
APD	0.5 (0.3–0.6)	0.6 (0.6–0.7)	0.03
LRD	1.5 (1.2–1.6)	1.1 (1.1–1.2)	0.30
CSA	0.7 (0.6–0.9)	0.7 (0.7–0.8)	0.88

Comparison of airway measurements with and without respiratory distress due to vascular ring and control

**Figure 2:**
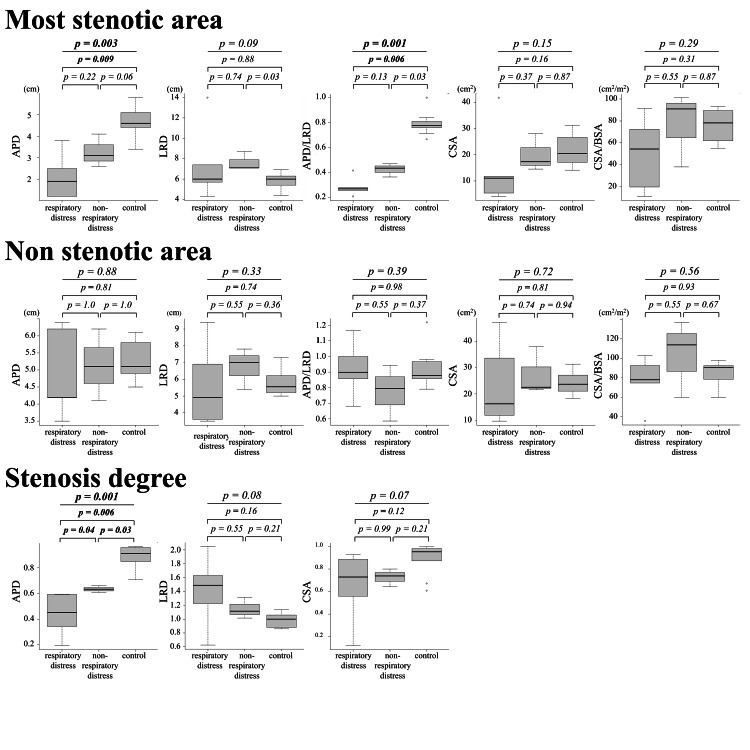
Comparative statistics and box-and-whisker plots of airway measurements stratified by the three groups: with and without respiratory distress by vascular ring and control Horizontal lines in boxes indicate medians, circles indicate outliers, and whiskers indicate data outside the 25-75th percentile range not considered outliers. APD, anterior-posterior diameter; BSA, body surface area; CSA, cross-sectional area; LRD, left-right diameter

Regarding airway measurements on contrast CT, as shown in Figure 2, there were significant differences in APD and APD/LRD at the most stenotic area, and SD-APD between the three groups (p = 0.003, 0.001, and 0.001, respectively). The results of the post hoc tests were as follows: APD at the most stenotic area showed a significant difference between the vascular ring with respiratory distress and control (p = 0.009). APD/LRD at the stenotic area with and without respiratory distress were significantly different from the control (p = 0.006 and 0.03, respectively). SD-APD was significantly different between all groups (p = 0.04 for vascular ring with and without respiratory distress).

## Discussion

A vascular ring surrounds the trachea and esophagus, forming a complete or partial stenosis, and resulting in clinical symptoms such as wheezing, stridor, and other forms of dyspnea and dysphagia [10]. We investigated the evaluation of the degree of airway stenosis by CT in pediatric patients diagnosed with vascular rings at our hospital. Several tracheal-related parameters were measured to assess tracheal compression. Our data show that cases with dyspnea had significantly smaller SD-APD, and cases with respiratory distress also tended to have smaller APD, but not significantly so. This means that when evaluating the airway, it is not only important to evaluate the most stenotic area but also vital to compare it with the non-stenotic area.

In infants, the trachea is especially vulnerable to exogenous factors and may develop dysplasia from prolonged compression, the extent of which greatly affects the prognosis; hence, early release of the vascular ring is desirable [11]. Additionally, vascular rings are often associated with or complicated by other diseases [10,12,13]. However, with the improved accuracy of prenatal diagnosis [14], it is essential to consider that some prenatally diagnosed vascular rings are clinically asymptomatic and so do not require surgical intervention. Therefore, diagnosis and treatment must be made with caution, based on integrated judgment from multiple tests and evaluation of airway compression is crucial.

In practice, echocardiography and angiography with CT or magnetic resonance imaging are the main imaging modalities used to confirm aortic arch abnormalities [15,16]. CT examination has the disadvantages of using ionizing radiation and nephrotoxic iodine-based contrast agents. However, compared to echocardiography and magnetic resonance imaging, CT examination provides a clearer view of the airway with higher resolution, more detailed information on cardiovascular structures in infants in a shorter time and with superior image quality, and they can visualize the airway without deep sedation [8,17]. Further, imaging in the early postnatal period-specifically before the closure of the ductus arteriosus-allows confirmation of its position. As the ductus arteriosus loses its contrast effect when it becomes cord-like after birth, this makes predicting its possible location difficult, and its positional relationship with the airway and esophagus remains unknown.

The finding of significant difference in SD-APD, rather than CSA stenosis degree, suggests that even if tracheal anatomy is deformed and the lumen has an oval or irregular shape and the cross-sectional area is preserved, a small APD may impede the passage of airflow and cause respiratory distress. Consideration of the severity of airway stenosis and prediction of prognosis, not limited to vascular rings, has been discussed from various perspectives. Anatomically, the evaluation of the stenosis rate in terms of tracheal diameter and the extent of stenosis in relation to the total length of the trachea are included. Cheng et al. [18] recommended conservative management of congenital tracheal stenosis by adding the condition that the diameter of the stenosis be greater than 60% of the normal diameter. In our study, respiratory distress was observed in patients with SD-APD less than 0.6, suggesting that respiratory distress may not be observed in patients with SD-APD of more than 0.6, even if airway stenosis is present. Furthermore, in a study including controls, SD-APD without respiratory distress ranged from 0.6 to 0.7, while controls ranged from 0.7 to 1.0. Thus, it may be possible to define a cut-off value for the presence or absence of both vascular rings and the presence of respiratory distress using SD-APD. Objective measures that include functional information in addition to anatomical assessment include airway resistance at the site of stenosis and changes in pressure and airflow pre- and post-stenosis [19]. In adults, a report has demonstrated intervention by directly inserting a pressure catheter into the airway and calculating airway resistance [20]. However, this method is highly invasive and difficult to perform in neonates and early infancy. Therefore, we present that evaluating the ratio before and after stenosis by comparing stenotic and non-stenotic areas, as in this study, could be a viable substitute.

Prenatal diagnosis was significantly less common among patients with respiratory distress. Prior to the widespread use of prenatal diagnosis, half of the symptomatic vascular ring cases were double aortic arch cases (more likely to cause symptoms) and half were right aortic arch cases with anomalous origin of the left subclavian artery (less likely to cause symptoms) [21]. Contrastingly, prenatal diagnosis was mostly the latter. This may be because prenatal diagnosis is made morphologically by fetal echocardiography regardless of the presence or absence of symptoms, whereas postnatal diagnosis is made mainly because vascular annulus is often suspected and diagnosed after respiratory symptoms appear. The number of cases of prenatal diagnosis of vascular rings is increasing; subsequently, the number of asymptomatic vascular ring cases is expected to increase in the future. Fetal ultrasound examination is minimally invasive and useful as a screening tool for vascular rings, and prenatal diagnosis allows parents to understand the specific risks and considerations in advance. Advantages include careful follow-up and early intervention when symptoms appear, even if the patient is asymptomatic at birth. While prenatal diagnosis allows for advanced preparation, it may also exaggerate symptoms and the need for surgery even before birth. Therefore, it is important to recognize that a prenatally diagnosed vascular ring does not mean that the patient is prone to respiratory distress. However, among vascular rings, a double aortic arch should be noted because prenatal diagnosis of a double aortic arch has been reported to be symptomatic [22]. Postnatal diagnosis is of course important, and contrast-enhanced CT at symptom onset is useful. However, screening all prenatally diagnosed patients with contrast-enhanced CT remains a radiation exposure issue, and the necessity of such an examination should be considered for every case. Worhunsky et al. proposed an algorithm for managing patients with a prenatal diagnosis of a vascular ring, emphasizing that patients with a vascular ring and significantly compressed trachea on CT cross-sectional images should be considered for selective repair, even if asymptomatic [23]. Our study suggests that SD-APD measurement may be useful as one of the indices to systematically assess the degree of tracheal compression on CT cross-sectional images.

The presence of congenital heart disease was not associated with the presence of respiratory distress. Approximately 12% of vascular rings are associated with cardiac disease [15]. In our study, all patients with cardiac complications had a ventricular septal defect, and two of them also had a left superior vena cava. Among the patients with ventricular septal defects, three patients had respiratory distress. One patient underwent only a ductus arteriosus transection, and the respiratory distress was relieved. The remaining two patients underwent ventricular septal defect closure and ductus arteriosus transection. Both patients had no pulmonary hypertension, and their heart failure was well controlled with diuretics for high pulmonary blood flow, with neither cardiac enlargement nor pulmonary congestion. Therefore, in all three cases of respiratory distress with ventricular septal defect, airway stenosis was considered the cause of the respiratory distress. Vascular ring surgery was not clearly related to the presence or absence of respiratory distress, and two cases were operated on despite presenting with no respiratory distress. In these two cases, the vascular ring release was performed simultaneously with the cardiac surgery. In the two cases in which neither respiratory distress nor congenital heart disease was present, no surgery was performed.

Genetic abnormalities were also not associated with the presence or absence of respiratory distress. All cases with genetic abnormalities had 22q11.2 deletion syndrome with congenital heart disease, and the classification of the vascular ring was IIIB1. The 22q11.2 deletion syndrome is a common chromosomal disorder characterized by a microdeletion in the region 11.2 on the long arm of chromosome 22, occurring approximately in 1 in 1,000 fetuses and 1 in 4,000 to 5,000 births. Moreover, 22q11.2 deletion syndrome is the second most common chromosomal abnormality causing congenital heart disease, after Down syndrome, and conus defects with abnormalities of the right aortic or interrupted aortic arch, or subclavian artery occur most frequently [24]. We found a high rate of 22q11.2 deletion syndrome in patients with vascular rings (44%). Other studies have reported a 5% incidence of 22q11.2 deletion and a 9% incidence of other chromosomal abnormalities [25,26]. The high rate of this genetic abnormality in our group and its association may be related to the fact that we included only patients with vascular rings. Once a diagnosis of a vascular ring is decided, families should be counseled about the high risk of genetic abnormalities to help them make decisions regarding genetic testing and pregnancy planning.

Limitations of this study are the small number of patients, bias in the classification of vascular ring cases, and its single-center, retrospective nature. Despite these limitations, our findings suggest an association between respiratory distress in the vascular ring and SD-APD on contrast-enhanced CT. Thus, we believe that it may be possible to define a cut-off value for SD-APD causing dyspnea, although further studies are needed to determine the generalizability of our results.

## Conclusions

In summary, our study reinforces our speculation that contrast-enhanced CT plays an important role in the diagnosis of vascular rings. Our results also represent the first publication to quantify airway stenosis, conventionally assessed visually-with several measures-and suggest that SD-APD of the trachea is a useful indicator for assessing the severity of airway stenosis in patients with vascular ring. Furthermore, it could be possible to define a cutoff value for SD-APD-causing dyspnea.
